# Supplementary material for: Does the Use of Local Antibiotics Affect Clinical Outcome of Patients with Fracture-Related Infection?
Source: Antibiotics (Basel). 2022 Sep 29;11(10):1330. doi: 10.3390/antibiotics11101330 (PMC9598690; doi:10.3390/antibiotics11101330)
Supplement: Supplementary file 1 [file antibiotics-11-01330-s001.zip › SupplementaryMaterials_TableS1_RecurrencePathogens - Copy.pdf]

Table S1: The causative pathogens of the initial and recurrent FRI of all patients with a recurrent infection.

No.	ALC used	Type of surgery performed	Pathogen(s) initial FRI	Pathogen(s) recurrent FRI	Identical pathogen(s) at initial and recurrent FRI (yes/no)
1	Yes	DAIR	*Escherichia coli*<br>*Enterococcus faecium*	*Escherichia coli*	Yes
2	Yes	DAIR	*Staphylococcus aureus*	*Staphylococcus aureus*	Yes
3	Yes	DAIR	*Enterobacter cloacae*	*Staphylococcus aureus*<br>*Staphylococcus epidermidis*<br>*Pseudomonas aeruginosa*	No
4	Yes	DAIR	*Staphylococcus aureus*<br>*Finegoldia magna*	*CN*	No
5	Yes	DAIR	*Streptococcus dysgalactiae*	*Streptococcus dysgalactiae*	Yes
6	Yes	DAIR	*Enterococcus faecalis*<br>*Enterobacter cloacae*	*Enterococcus faecalis*	Yes
7	Yes	External Fixation	*Staphylococcus aureus*<br>*Enterococcus faecalis*	*CN*	No
8	Yes	External Fixation	*Staphylococcus aureus*	*Staphylococcus aureus*	Yes
9	Yes	External Fixation	*Staphylococcus aureus (MRSA)*	*Staphylococcus aureus (MSSA)*	No
10	Yes	External Fixation	*Streptococcus pyogenes*<br>*Staphylococcus aureus*	*CN*	No
11	Yes	External Fixation	*Escherichia coli*<br>*Staphylococcus aureus*<br>*Streptococcus dysgalactiae*	*Escherichia coli*	Yes
12	Yes	Internal Fixation	*Enterococcus caselflavious*	*CoNS*	No
13	Yes	Internal Fixation	*CN*	*Enterobacter cloacae*	No
14	Yes	Removal	*Escherichia coli*<br>*Klebsiella oxytoca*<br>*Dermabacter hominis*	*Finegoldia magna*	No
15	Yes	Removal	*Staphylococcus aureus*	*Staphylococcus aureus*	Yes
16	Yes	Removal	*Staphylococcus aureus*<br>*Morganella morganii*	*Streptococcus agalactiae*	No
17	Yes	Removal	*Escherichia coli*<br>*Enterococcus faecalis*<br>*Mixed anaerobes*	*Staphylococcus aureus*<br>*Morganella morganii*<br>*Actinomyces spp.*<br>*Corynebacterium spp.*	No
18	Yes	Removal	*Staphylococcus epidermidis*	*CN*	No
19	Yes	Removal	*Staphylococcus aureus*	*Staphylococcus aureus*	Yes
20	Yes	Removal	*Staphylococcus aureus*	*CN*	No
21	Yes	Removal	*CN*	*Klebsiella oxytoca*<br>*Enterococcus faecalis*	No
22	Yes	Removal	*Staphylococcus aureus*	*Staphylococcus aureus*	Yes
23	Yes	Removal	*Staphylococcus epidermidis*<br>*Enterobacter cloacae*	*Staphylococcus aureus*<br>*Bacteroides spp.*<br>*Stenotrophomonas spp.*<br>*Anaerococus spp.*	No
24	Yes	Removal	*Staphylococcus aureus*<br>*Escherichia coli*<br>*Enterococcus faecalis*<br>*Proteus mirabilis*	*CN*	No

25	Yes	Removal	*CN*	*Staphylococcus aureus*	No
26	No	DAIR	*Corynebacterium amycolatum*<br>*Corynebacterium urealyticum*	*CN*	No
27	No	DAIR	*Staphylococcus aureus*	*Pseudomonas aeruginosa*	No
28	No	DAIR	*Staphylococcus aureus*<br>*Finegoldia magna*	*Staphylococcus aureus*	Yes
29	No	DAIR	*Staphylococcus aureus*<br>*Finegoldia magna*<br>*Peptoniphilus harei*	*Proteus mirabilis*<br>*Klebisella pneumoniae*<br>*Morganella morganii*	No
30	No	DAIR	*Staphylococcus lugdunensis*<br>*Staphylococcus epidermidis*<br>*Streptococcus agalactiae*	*Staphylococcus lugdunensis*<br>*Cutibacterium acnes*	Yes
31	No	DAIR	*Pasteurella canis*<br>*Peptostreptococcus canis*	*Staphylococcus lugdunensis*<br>*Corynebacterium auriscanis*<br>*Enterobacter cloacae*<br>*Staphylococcus pseudointermedius*<br>*Acinetobacter baumanii*<br>*Staphylococcus epidermidis*	No
32	No	DAIR	*Staphylococcus aureus*	*Staphylococcus aureus*<br>*Finegoldia magna*	Yes
33	No	DAIR	*Staphylococcus aureus*	*CN*	No
34	No	DAIR	*Rhodococcus hoagii (equi)*	*CN*	No
35	No	DAIR	*Staphylococcus aureus*	*Staphylococcus hominis*<br>*Paenibacillus species*	No
36	No	DAIR	*Staphylococcus aureus*<br>*Staphylococcus caprae*<br>*Corynebacterium amycolatum*	*Staphylococcus epidermidis*<br>*Escherichia coli*	No
37	No	DAIR	*Serratia marcescens*<br>*Staphylococcus epidermidis*<br>*Peptoniphilus gorbachii*	*Serratia marscesens*<br>*Streptococcus dysgalactiae*	Yes
38	No	DAIR	*Staphylococcus aureus*<br>*Porphyromonas uenonis*<br>*Prevotella bivia*<br>*Dialister micraerophilus*	*Staphylococcus aureus*<br>*Streptococcus mitis*<br>*Actinomyces odontolyticus*<br>*Prevotella melaninogenica*<br>*Parvimonas micra*<br>*Veillonella parvula*	Yes
39	No	DAIR	*Staphylococcus aureus*	*Staphylococcus haemolyticus*	No
40	No	DAIR	*Staphylococcus aureus*	*Escherichia coli*	No
41	No	DAIR	*Staphylococcus aureus*<br>*Peptostreptococcus anaerobius*	*Haemophilis parainfluenza*<br>*Staphylococcus epidermidis*	No
42	No	DAIR	*Actinomyces neuii*	*Dermabacter hominis*<br>*Cutibacterium acnes*	No
43	No	DAIR	*Staphylococcus aureus*<br>*Streptococcus pyogenes*	*Staphylococcus hominis*	No
44	No	DAIR	*CN*	*Stretococcus auginosus milleri*	No
45	No	DAIR	*Staphylococcus aureus*<br>*Micrococcus luteus*	*CN*	No
46	No	DAIR	*Staphylococcus lugdunensis*<br>*Corynebacterium simulans*	*Staphylococcus hominis*<br>*Staphylococcus equorum*<br>*Kocura rhizophilia*<br>*Micrococcus luteus*	No
47	No	DAIR	*Streptococcus dysgalactiae*	*CN*	No

48	No	DAIR	*Staphylococcus aureus*<br>*Cutibacterium acnes*<br>*Streptoccus mitis*<br>*Finegoldia Magna*<br>*Staphylococcus lugdenensis*	*Staphylococcus aureus*<br>*Cutibacterium acnes*	Yes
49	No	DAIR	*Staphylococcus aureus*	*CN*	No
50	No	External Fixation	*Staphylococcus aureus*	*CN*	No
51	No	External Fixation	*CN*	*Staphylococcus epidermidis*	No
52	No	Internal Fixation	*Staphylococcus epidermidis*<br>*Staphylococcus capitis*	*Staphylococcus epidermidis*<br>*Cutibcaterium acnes*	Yes
53	No	Removal	*Staphylococcus epidermidis*	*Streptococcus spp.*	No
54	No	Removal	*Staphylococcus aureus*	*Staphylococcus epidermidis*<br>*Streptococcus agalactiae*<br>*Finegoldia magna*	No
55	No	Removal	*Finegoldia magna*<br>*Staphylococcus lugdunensis*	*Staphylococcus aureus*	No
56	No	Removal	*CN*	*CN*	No
57	No	Removal	*Staphylococcus epidermidis*<br>*Pseudomonas aeruginosa*	*Staphylococcus epidermidis*<br>*Pseudomonas aeruginosa*	Yes
58	No	Removal	*Escherichia coli*<br>*Enterococcus faecalis*<br>*Staphylococcus aureus*<br>*Finegoldia magna*	*Escherichia coli*	Yes
59	No	Removal	*Staphylococcus epidermidis*<br>*Corynebacterium tuberculostearicum*<br>*Staphylococcus aureus*	*Staphylococcus epidermidis*	Yes

CoNS: coagulase negative staphylococci; DAIR: debridement, antibiotics and implant retention; CN: culture negative; spp.: species.
Identical pathogen, isolated at the initial FRI treatment and at the time of recurrence, were found in 10/34 (29.4%) of cases in the non-ALC group and in 9/25 (36.0%) of cases in the ALC group.
