# Supplementary figures and images for: Does the Use of Local Antibiotics Affect Clinical Outcome of Patients with Fracture-Related Infection?
Source: Antibiotics (Basel). 2022 Sep 29;11(10):1330. doi: 10.3390/antibiotics11101330 (PMC9598690; doi:10.3390/antibiotics11101330)

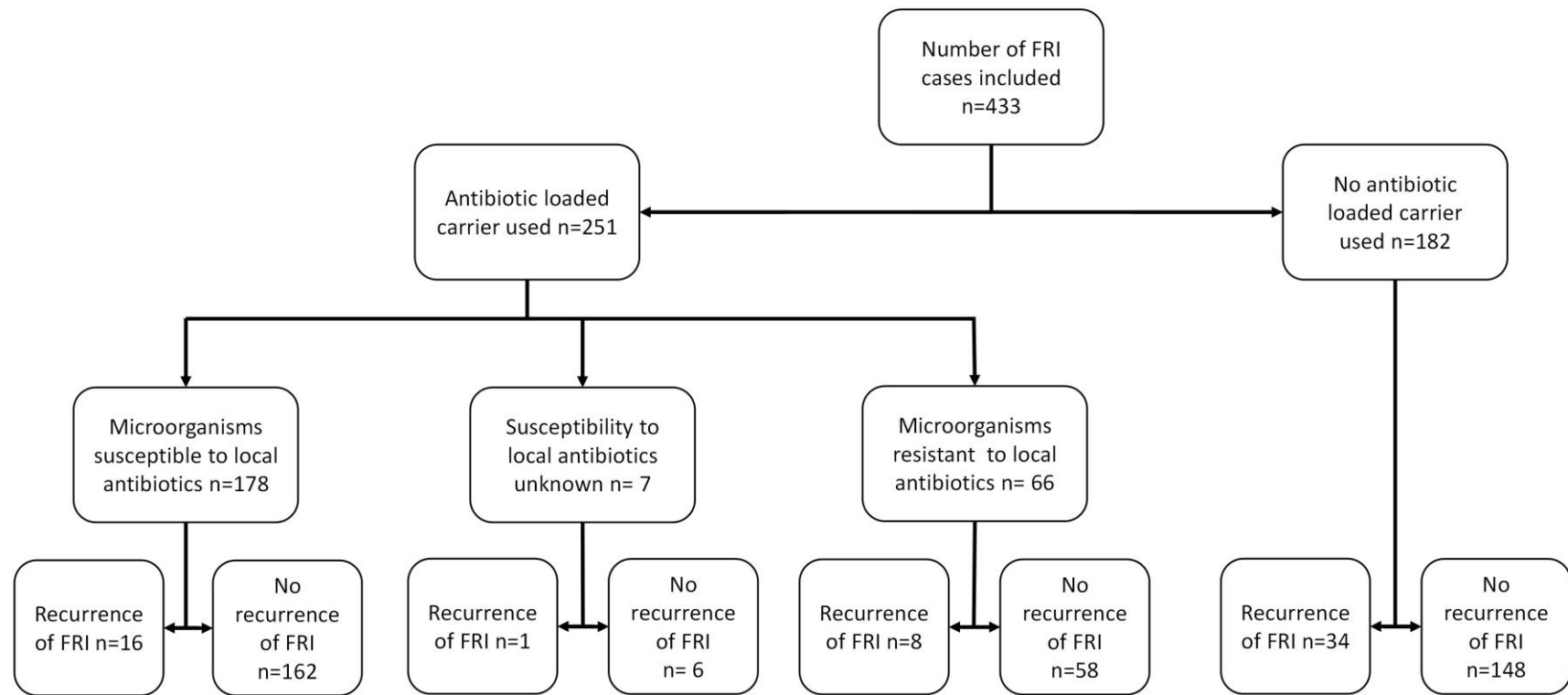

Figure S1. A flowchart of the outcome of FRI according to the use of- and susceptibility to ALCs.

Supplement: Supplementary file 1 [file antibiotics-11-01330-s001.zip › SupplementaryMaterials_Figure S1_Flowchart.pdf]
